# Nuclear p53-mediated repression of autophagy involves PINK1 transcriptional down-regulation

**DOI:** 10.1038/s41418-017-0016-0

**Published:** 2018-01-19

**Authors:** Thomas Goiran, Eric Duplan, Lila Rouland, Wejdane el Manaa, Inger Lauritzen, Julie Dunys, Han You, Frédéric Checler, Cristine Alves da Costa

**Affiliations:** 1 Université Côte d’Azur, INSERM, CNRS, IPMC, team labeled “Laboratory of Excellence (LABEX) Distalz”, 660 route des Lucioles, 06560 Sophia-Antipolis, Valbonne France; 2 State Key Laboratory of Cellular Stress Biology, Innovation Center for Cell Signaling network, School of Life Sciences, Xiamen University, Xiamen, Fujian 361102 China

## Abstract

p53 is a transcription factor that is implicated in the control of both apoptotic and autophagic cell death. This tumor suppressor elicits both pro-autophagic and anti-autophagic phenotypes depending of its intracellular localization. The ability of p53 to repress autophagy has been exclusively associated to its cytoplasmic localization. Here, we show that transcriptional activity of p53 also contributes to autophagy down-regulation. Thus, nuclear p53 controls PINK1, a key protein involved in the control of mitophagy, by repressing its promoter activity, protein and mRNA levels, *ex-vivo* and *in vivo*. We establish that deletion of an identified p53 responsive element on *PINK1* promoter impacts p53-mediated *PINK1* transcriptional repression and we demonstrate a p53-*PINK1* physical interaction by chromatin immunoprecipitation. Accordingly, we show that only nuclear p53 accounts for its ability to repress *PINK1* gene transcription. Further, we demonstrate *ex-vivo* and *in vivo* that p53 invalidation in human cells increases LC3 maturation as well as optineurin and NDP52 autophagy receptors expression and down-regulates TIM23, TOM20 and HSP60 mitophagy markers. Importantly, this phenotype is mimicked by *TP53* invalidation in mice brain. Finally, by combining pharmacological and genetic approaches, we show that the p53-mediated negative regulation of autophagy is PINK1-dependent. Thus pifithrin-α-mediated blockade of p53 transcriptional activity enhances LC3 maturation and reduces p62, TIM23, TOM20 and HSP60 protein levels. This pifithrin-α-associated pro-mitophagy phenotype is fully abolished by PINK1 depletion. This data unravels a novel pathway by which nuclear p53 can repress autophagy/mitophagy that could underlie important dysfunctions in both neurodegenerative and cancer diseases.

## Introduction

p53 is a key multifunctional protein, the implication of which has been extensively investigated in various pathologies. It is a transcription factor that may positively or negatively regulate a huge number of genes [1] involved in cell-fate control mechanisms linked to both apoptosis and autophagy. There is a consensus concerning the fact that p53-dependent control of autophagy is directly driven by its subcellular localization [2]. Thus, in stress conditions, p53 acts as a pro-autophagic mediator [2, 3] by modulating genes implicated in the regulation of mTOR (mammalian Target Of Rapamycine), a negative modulator of autophagy [4]. p53 also controls the transcription of Death-Associated Protein Kinase-1 [5] and Damage-Regulated Autophagy Modulator [6], two key players of autophagic response. In contrast to this transcriptional factor mediated pro-autophagic phenotype, cytoplasmic p53 can repress autophagy in absence of any cellular stress [7, 8]. This inhibitory effect is independent of its transcriptional function and molecularly linked to the activation of the AMPK-dependent inhibition of mTOR signaling cascade [7].

Interestingly, several works have shown a functional interplay between p53 and parkin. Thus, in physiological conditions, parkin and p53 control the transcription of each other via their DNA binding properties [9–11]. Parkin is also involved in the degradation of several toxic proteins by the proteasome via its ubiquitin-ligase activity [12] and was shown to be recruited to mitochondria and activated by PINK1 (PTEN-Induced Kinase 1) upon its phosphorylation [13, 14]. In pathological conditions, the turnover of PINK1 is inhibited, leading to its stabilization in mitochondria outer membrane and parkin recruitment and phosphorylation. Consequently, activated parkin mediates the specific elimination of defective mitochondria by the lysosomal system [15, 16]. These data linking p53 and parkin led us to hypothesize that p53 and PINK1 could be linked at a molecular level to control mitophagy.

We describe here the first pro-autophagic gene transcriptionally controlled by p53 in both basal and stress conditions. We demonstrate that p53 represses the transcription of the pro-mitophagic gene *PINK1* by several approaches. Thus, pharmacological and genetic modulation of p53 leads to a down-regulation of *PINK1* transcription *ex-vivo* and *in vivo*. This regulation is mediated by p53 physical interaction with *PINK1* promoter and strictly dependent of p53 nuclear subcellular localization. Finally, we show that p53-induced repression of autophagy is PINK1-dependent.

Thus, our study delineates for the first time an anti-autophagic phenotype implying p53 transcriptional activity in basal and stress conditions. This novel observation may have a major impact in multiple diseases including neurodegenerative disorders and cancer where p53 function is altered and where autophagic dysfunction has been consistently documented.

## Results

### Pharmacological modulation of p53 impacts *PINK1* transcription

**Fig. 1:**
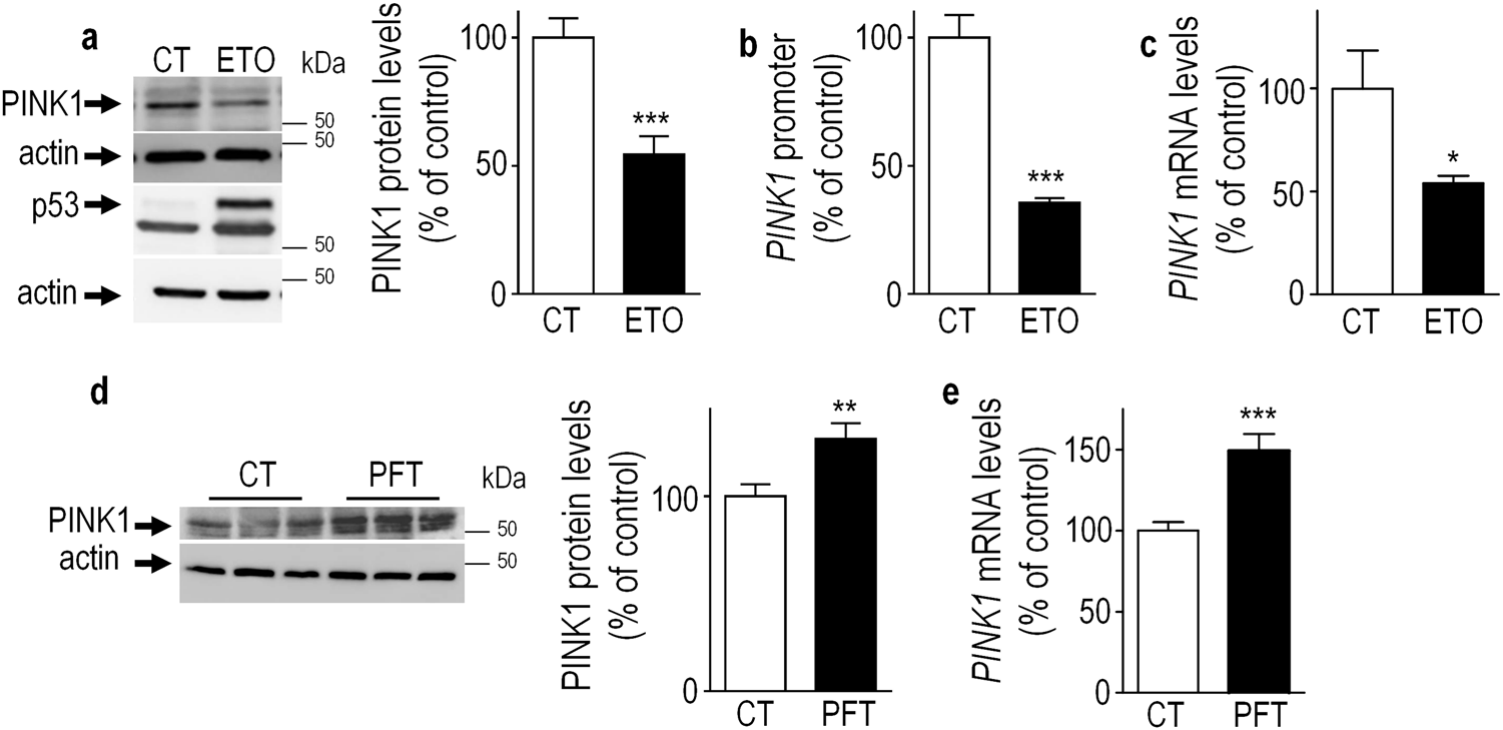
Pharmacological modulation of p53 impacts *PINK1* transcription. SH-SY5Y cells were treated with vehicle (CT), etoposide (ETO, 150 µM, 16 h) or pifithrin-alpha (PFT, 10 µM, 16 h) then PINK1 protein (**a**,**d**, *N* = 9), promoter activity (**b**, *N* = 9) and mRNA levels (**c**,**e**, *N* = 6) were analyzed as described in the Methods section. p53 and actin immunoreactivities are provided as read-out of p53 activation and gel loading controls (**a**, **d**, respectively). Bars represent the means ± SEM of 3 independent experiments performed in triplicate (**a**, **b**, **d**) or duplicates (**c**,**e**) and are expressed as percentage of vehicle-treated control cells. Statistical analyses were performed with GraphPad Prism software by using unpaired Student’s t-test. Significant differences are: *p < 0.05, **p < 0.01, ***p < 0.001.

We have modulated p53 expression by treating SH-SY5Y neuroblastoma human cells with etoposide (ETO), a topoisomerase II genotoxic agent that stabilizes p53 by favoring its phosphorylation [17]. As expected, we observed an ETO-induced increase in p53 expression (Fig. 1a) that was associated with a reduction of PINK1 protein expression (Fig. 1a), promoter transactivation (Fig. 1b) and mRNA levels (Fig. 1c), suggesting a p53-dependent inhibitory control of *PINK1* transcription. We confirmed this hypothesis by assessing the effect of pifithrin-α (PFT), a chemical inhibitor of p53 that specifically blocks its transcriptional activity [18]. Thus, PFT-induced blockade of p53 activity increases both PINK1 protein expression (Fig. 1d) and mRNA levels (Fig. 1e). Overall, this set of data identifies p53 as a transcriptional inhibitor of *PINK1* transcription.

### Endogenous and overexpressed p53 repress *PINK1* transcription

**Fig. 2:**
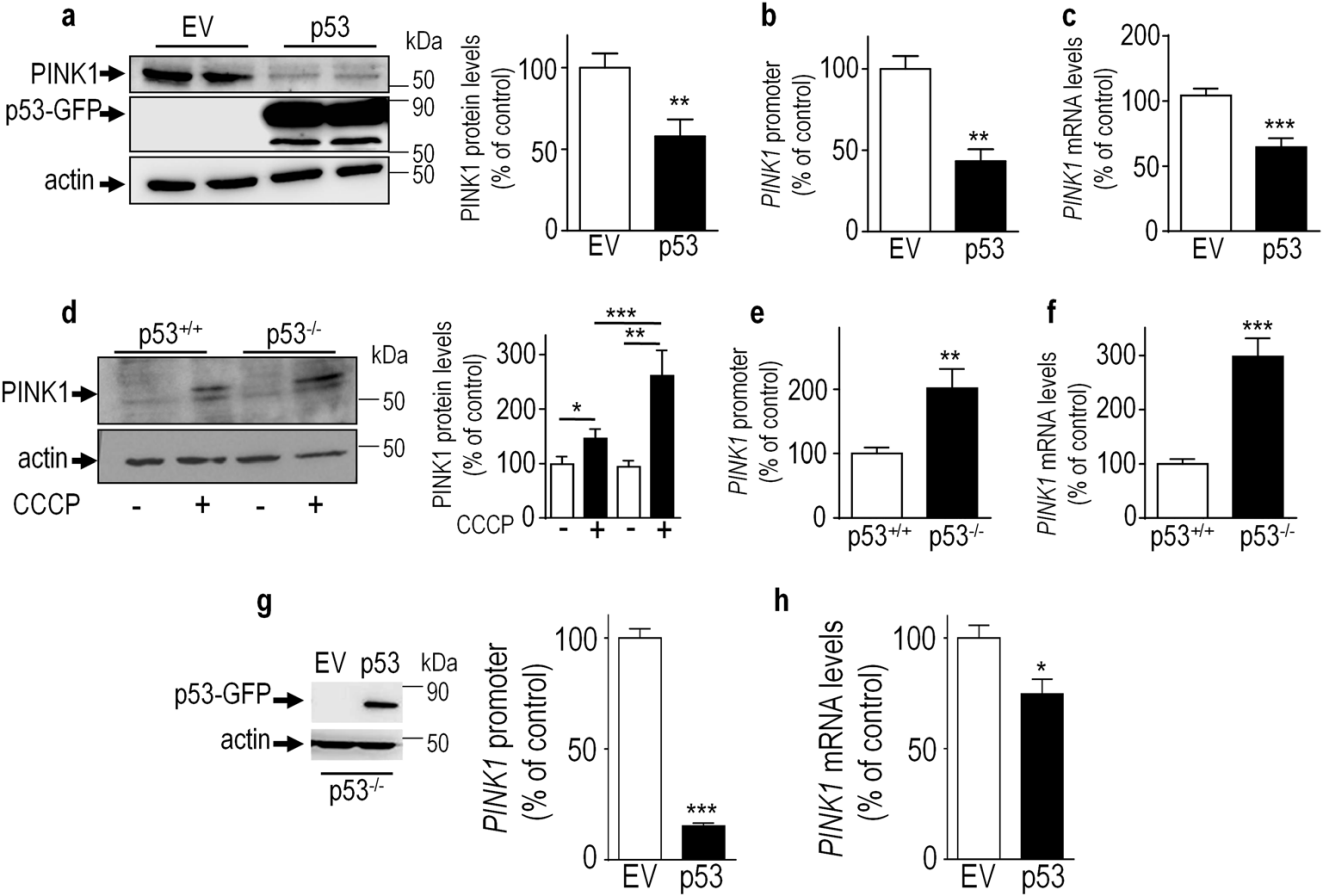
Overexpressed and endogenous p53 repress *PINK1* transcription. SH-SY5Y cells transiently transfected with an empty vector (EV, white bars) or wild-type p53 cDNA (p53, black bars) were assessed for PINK1 protein (**a**, *N* = 8), promoter activation (**b**, *N* = 6) and mRNA levels (**c**, *N* = 12). PINK1 protein levels in basal (−, white bars) and CCCP ( + , black bars, 10 µM/6 h) stress conditions were analyzed in control (p53^+/+^, black bars) or p53-deficient (p53^−/−^, white bars) HCT116 cells (**d**, *N* = 12). *PINK1* promoter transactivation (**e**, *N* = 6) and mRNA levels (**f**, *N* = 12) were analyzed in control (p53^+/+^, black bars) or p53-deficient (p53^−/−^, white bars) HCT116 cells in basal conditions as described in the Methods section. p53 and actin protein levels are provided in **a** and **d** as transfection and protein charge controls. HCT116 p53-deficient (p53^−/−^) cells transiently transfected with an empty vector (EV, white bars) or wild-type p53 cDNA (p53, black bars) were assessed for p53 expression (**g**, left panel) *PINK1* promoter transactivation (**g**, *N* = 8) and mRNA levels (**h**, *N* = 6). Bars represent the means ± SEM of 3 independent experiments performed in duplicates (**a**,**b**,**e**,**g**,**h**) or quadruplicates (**c**,**d**,**f**) and are expressed as percentage of control EV (**a**-**c**, **g**,**h**) or p53^+/+^ cells (**d**-**f**). Statistical analyses were performed with GraphPad Prism software by using unpaired student’s *t*-test. Significant differences are: *p < 0.05,**p < 0.01, and ****p < 0.0001.

We aimed at examining more directly the influence of overexpressed and endogenous p53 on *PINK1* transcription. Transient transfection of human wild-type p53 cDNA in SH-SY5Y cells led to drastic reductions of PINK1 protein, promoter activity and mRNA levels (compare control empty vector, (EV, white bars) and p53 (black bars) in Fig. 2a-c). The assessment of PINK1 modulation by endogenous p53 was examined in the human colorectal carcinoma cell line HCT116, where *TP53* gene was genetically invalidated. We confirmed the general observations that endogenous PINK1 expression is generally low in fibroblasts and, more generally, in non-neuronal cells (See Fig. 2d, p53^+/+^ lane -). Thus, in this set of experiments, we took advantage of previous data, which consistently documented a stabilization of PINK1 by the stress-associated inducer of mitochondrial-depolarization—[2-(3-chlorophenyl) hydrazinylyidene] propanedinitrile (CCCP) [19]. As expected, PINK1 expression is indeed increased by CCCP in HCT116 cells (compare-and +CCCP conditions in p53^+/+^ lanes in Fig. 2d). Interestingly, p53 depletion (p53^−/−^) increases PINK1 protein expression, promoter transactivation and mRNA levels (Fig. 2d-f, black bars). In rescue experiments, we show that human p53 cDNA transfection in p53^−/−^ HCT116 cells restore p53-associated inhibitory effect on *PINK1* promoter activity (Fig. 2g) and mRNA levels (Fig. 2h). Similar phenotype was observed in an additional model of *TP53* invalidation (Immortalized mouse embryonic fibroblasts invalidated for either *p19*^*arf*^ (A-, empty bars) or for both *TP53* and *p19*^*arf*^ genes (AP-, black bars)) [20], which display increased levels of *PINK1* promoter activity (Fig. S1a) and mRNA levels (Fig. S1b). Altogether, combined genetic and pharmacological data demonstrate the role of endogenous p53 as a transcriptional repressor of PINK1.

### p53 controls *PINK1* transcription and protein expression ***in vivo***

**Fig. 3:**
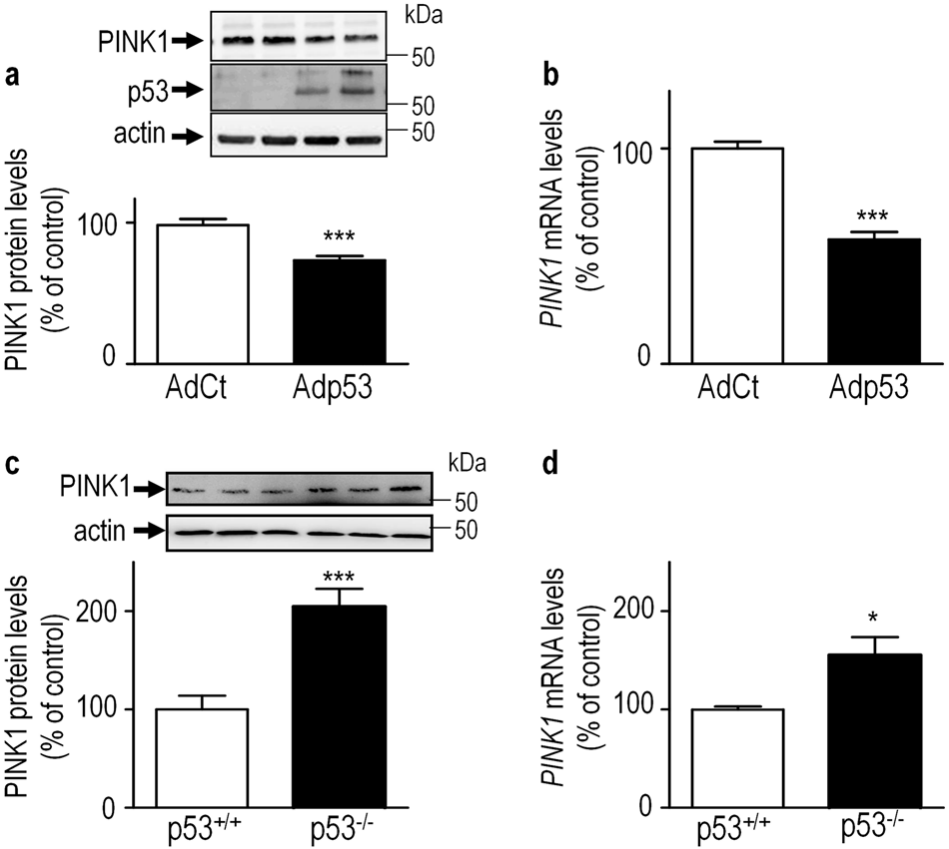
p53 controls PINK1 transcription *in vivo*. (**a**,**b**) Effect of adenovirus-mediated wild-type p53 (Adp53, black bars) overexpression on PINK1 protein (**a**, *N* = 8) and mRNA (**b**, *N* = 8) levels compared to control virus (AdCt, white bars) *in vivo* as described in the Methods. Analyses of PINK1 protein (**c**, *N* = 6) and mRNA levels (**d**, *N* = 8) in control (p53^+/+^, white bars) or *TP53* gene invalidated (p53^−/−^, black bars) in mouse brain samples as described in the Methods. Representative gels of PINK1, p53 (virus transduction efficiency) and actin (protein charge control) protein levels are provided in upper panels **a** and **c**. Bars represent the means ± SEM of 2 independent experiments performed in quadruplicates and are expressed as percentage of control (AdCt, p53^+/+^) mouse brain samples. Bars represent the means ± SEM of 3 or 4 independent experiments performed in duplicate (**c**,**d**, respectively) and are expressed as percentage of control (AdCt, p53^+/+^) mouse brain samples. Statistical analyses were performed with GraphPad Prism software by using unpaired Student’s t-test. Significant differences are: *p < 0.05, and ***p < 0.001.

Is PINK1 also down-regulated by p53, *in vivo*? We have analyzed the impact of both adenoviral- overexpression (Fig. 3a, b) or invalidation (Fig. 3c, d) of p53 on PINK1 protein and mRNA levels in mice brains. Striatal overexpression of p53 decreases PINK1 protein (Fig. 3a) and mRNA (Fig. 3b) levels. Conversely, depletion of endogenous p53 increases PINK1 protein (Fig. 3c) and mRNA (Fig. 3d) levels. It should be noted that p53 invalidation impacts neuronal PINK1 transcription and protein expression, *in vivo*, in absence of any stress. This data confirms the fact that endogenous level of PINK1 protein remains under thresholds of detection in non-neuronal cellular models (thus requiring CCCP treatment to unravel its expression, Fig. 2a) but not in a neuronal context. Overall, our data show that endogenous p53 negatively controls PINK1 protein and mRNA levels *in vivo*, thus confirming our observations gathered in various cellular models (Figs. 1 and 2).

### Identification and validation of a p53 binding site on *PINK1* promoter

**Fig. 4:**
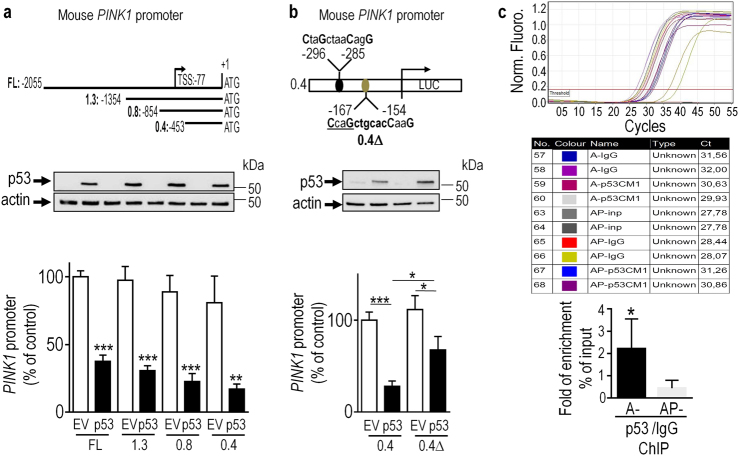
Mapping and validation of p53 binding domain of PINK1 promoter. Upper panel **a** (*N* = 12) represents the 5’end deletion constructs of the full-length (FL) mouse PINK1 promoter region. The PINK1 promoter constructs described in **a** (upper scheme) were co-transfected with the β-galactosidase reporter gene and either empty vector (EV, white bars) or wild-type p53 (p53, black bars) cDNA in HEK293 cells and then luciferase activity was measured as described in Methods. Upper panel **b** represents the two potential p53-responsive elements (-296/-285 and -167/-154) on the shorter 0.4Kb-luciferase PINK1 promoter construct. In lower panel (**b**, *N* = 12), PINK1 promoter constructs 0.4Kb wild-type (0.4) or deleted from the -167/-164 sequence (0.4∆) were co-transfected with the β-galactosidase reporter gene and either empty cDNA (EV, white bars) or wild-type p53 (p53, black bars) cDNA, then luciferase activity was measured as described in Methods. Representative gels of p53 expression and actin as transfection efficiency and gel charge controls are provided in **a** and **b**. Bars represent the means ± SEM of 4 independent experiments performed in triplicates and are expressed as percentage of control (FL, 0.4) EV transfected cells. Upper panel **c** represents real-time PCR curves of samples from a standard ChIP experience (**c**, middle panel) performed in control (A-, black bars) and *TP53*-invalidated MEF cells (AP-, gray bars). Lower panel **c** represents the quantification analysis of the ChIP enrichment of PINK1 promoter using and anti-p53 antibody (anti-CM1) over that of control IgG. Bars represent the means ± SEM of 2 independent experiments performed in triplicate and are expressed as fold of enrichment according to the formula described in the methods. Statistical analyses were performed with GraphPad Prism software by using unpaired Student’s *t*-test. Significant differences are: *p < 0.05, **p < 0.01, ***p < 0.001.

In order to map the putative physical domain of interaction between p53 and *PINK1* promoter, we have analyzed the effect of p53 expression (see Fig. 4a, middle) on full-length (FL) and 5′end-truncated constructs of *PINK1* mouse promoter driving luciferase expression (Fig. 4a, upper). p53 represses *PINK1* transcription whatever the promoter construct used (Fig. 4a, lower), indicating that at least one putative functional p53 consensus binding motif was located in the shorter 0,4 kb construct. *In silico* examination of this sequence, led to the identification of at least two putative (−296/−285 and −167/−154) p53 responsive elements (Fig. 4b, upper). Since the −167/−154 (***CCAG***ctgcac***CAAG)*** sequence fits better with the canonical consensus p53 responsive element (CxxG-N6-CxxG), we have deleted the −167/−164 CCAG motif from the 0.4 kb sequence (construct referred to as 0.4Δ) and examined the impact of this deletion on p53-dependent *PINK1* promoter activity. This deletion significantly reduced the p53-dependent decrease of *PINK1* promoter activity observed with wild-type 0.4 construct (compare black bars in Fig. 4b, lower). This data validates the −167/−154 sequence as a functional p53 responsive element and confirms the ability of p53 to directly repress *PINK1* transcription. However, this deletion did not fully abolish p53-associated inhibitory effect (Fig. 4b, lower). This could indicate a potential participation of the less conserved responsive element located at −296/−285. To validate the physical interaction of p53 with the -167/-154 *PINK1* promoter region in physiological conditions, we have performed chromatin immunoprecipitation assay (ChIP). Real-time PCR assessment (Fig. 4c upper) of ChIP enrichment of *PINK1* promoter DNA using specific p53 immunoprecipitant antibodies (CM1, Fig. 4c middle) and negative control IgG in both control (A-) and p53 (AP-) invalidated cells indicates a physical interaction of p53 with *PINK1* promoter in cells harboring *TP53* (A-), but not in cells devoid of *TP53* (AP-, Fig. 4c, lower).

### Repression of *PINK1* transcription requires nuclear localization of p53

**Fig. 5:**
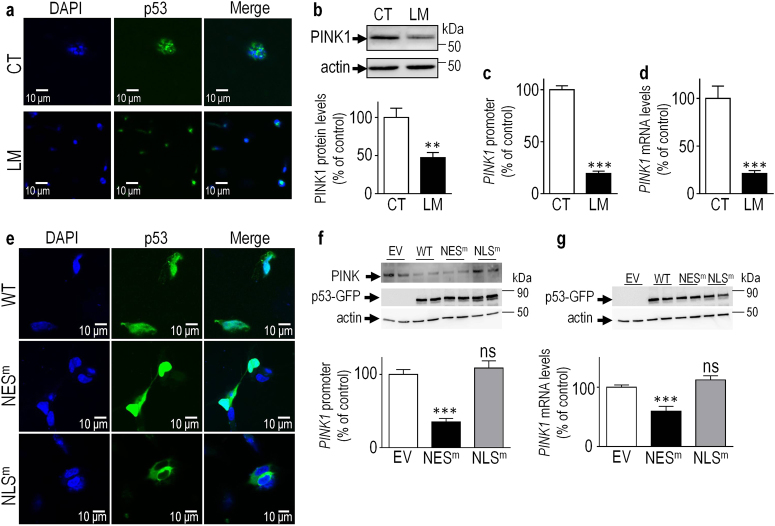
Impact of the pharmacological and genetic modulation of p53 localization on *PINK1* transcription. (**a**) Immunohischemical detection of endogenous p53 and DAPI nuclear labeling in SH-SY5Y cells treated with vehicle (CT) or leptomycin (LM) as described in the methods. (**b-d**) PINK1 protein expression (**b**,**f**, *N* = 12), promoter activation (**c**, *N* = 6) and mRNA levels (**d**, *N* = 9) were analyzed in SH-SY5Y cells treated with vehicle (CT) or with leptomycin (LM) as described in the Methods. (**e**) Immunohistochemical analysis of GFP-tagged p53 constructs versus DAPI nuclear labeling showing the presence of wild-type p53 (WT) in the nucleus and cytoplasm, p53 NES mutant (L22Q, W23S p53, NES^m^) in the nucleus and p53 NLS mutant (K280-282A p53, NLS^m^) in the cytoplasm. (**f**,**g**) SH-SY5Y cells transiently transfected with empty vector (EV), wild-type p53 (WT), p53 NES mutant (NES^m^), or p53 NLS mutant (NLS^m^) and then assessed for PINK1 promoter activity (**f**, *N* = 12) and mRNA levels (**g**, *N* = 12). p53 (**f**,**g**) and actin (**b**,**f**,**g**) immunoreactivities are provided as read-out of p53 activation and gel loading respectively. Bars represent the means ± SEM of 3 independent experiments performed in duplicates (**c**) or quadruplicates (**b**,**f**,**g**) and are expressed as percentage of vehicle-treated cells (**b-d**) or EV control cells (**e-g**). Statistical analyses were performed with GraphPad Prism software by using unpaired Student’s *t*-test. Significant differences are: **p < 0.01, ****p < 0.0001 and ns, non-significant.

As stated in the introduction, sub-cellular localization of p53 conditions its transcription factor properties and by consequence its pro- or anti-autophagic function [2]. We have investigated the impact of the pharmacological and genetic modulation of p53 subcellular localization on *PINK1* transcription. Leptomycin B (LM), specifically and potently inhibits the CRM1/exportin 1 pathway of nuclear export by directly binding the CRM1 protein [21, 22] and was shown to block p53 in the nucleus [23]. The impact of LM on p53 translocation was confirmed in Fig. 5a by immunofluorescence imaging. Thus, as expected, LM treatment blocks p53 in the nucleus as evidenced by the exclusive colocalization between p53 and nucleus marker DAPI (compare merge DAPI/p53 in control (CT) versus LM conditions). Importantly, LM treatment triggers a drastic reduction of PINK1 protein expression (Fig. 5b), promoter activity (Fig. 5c) and mRNA levels (Fig. 5d), indicating that the control of *PINK1* transcription is linked to p53 nuclear localization. Several works have shown that nuclear p53 exerts a pro-autophagic function while cytoplasmic p53 triggers an anti-autophagic phenotype. Thus, we were facing a case where PINK1 could well behave as the first pro-autophagic gene transcriptionally repressed by nuclear p53. To strengthen this hypothesis, we designed constructs encoding either wild-type p53 coupled to GFP (WT) or harboring two mutations in its previously delineated nuclear export (GFP-p53 L22Q, W23S, NES^m^) or nuclear import (GFP-p53 KKK280-282AAA, NLS^m^) domains [23, 24] and we examined their impacts on *PINK1* transcriptional regulation in SH-SY5Y cells. Immunohistochemical analyses of GFP-tagged p53 constructs confirmed that the NES and NLS mutations indeed blocked p53 in the nucleus (Fig. 5e, middle**)** and cytoplasm (Fig. 5e, lower**)**, respectively. Of most interest, nuclear p53 decreased PINK1 expression (Fig. 5f, upper) *PINK1* promoter activation and mRNA levels (NES^m^, black bars in Fig. 5f, g) while sequestration of p53 in the cytoplasmic compartment fully abolished p53-induced reductions of PINK1 protein expression (Fig. 5f upper), promoter activity and mRNA levels (NLS^m^, gray bars in 5f,g). This data firmly demonstrates that *PINK1* transcriptional repression requires exclusive nuclear localization of p53 and thus, unravels PINK1 as the first pro-autophagic gene transcriptionally repressed by p53. This is the very first demonstration of an anti-autophagic phenotype linked to nuclear p53 transcription factor function.

### p53 represses mitophagy *ex-vivo* and *in vivo*

**Fig. 6:**
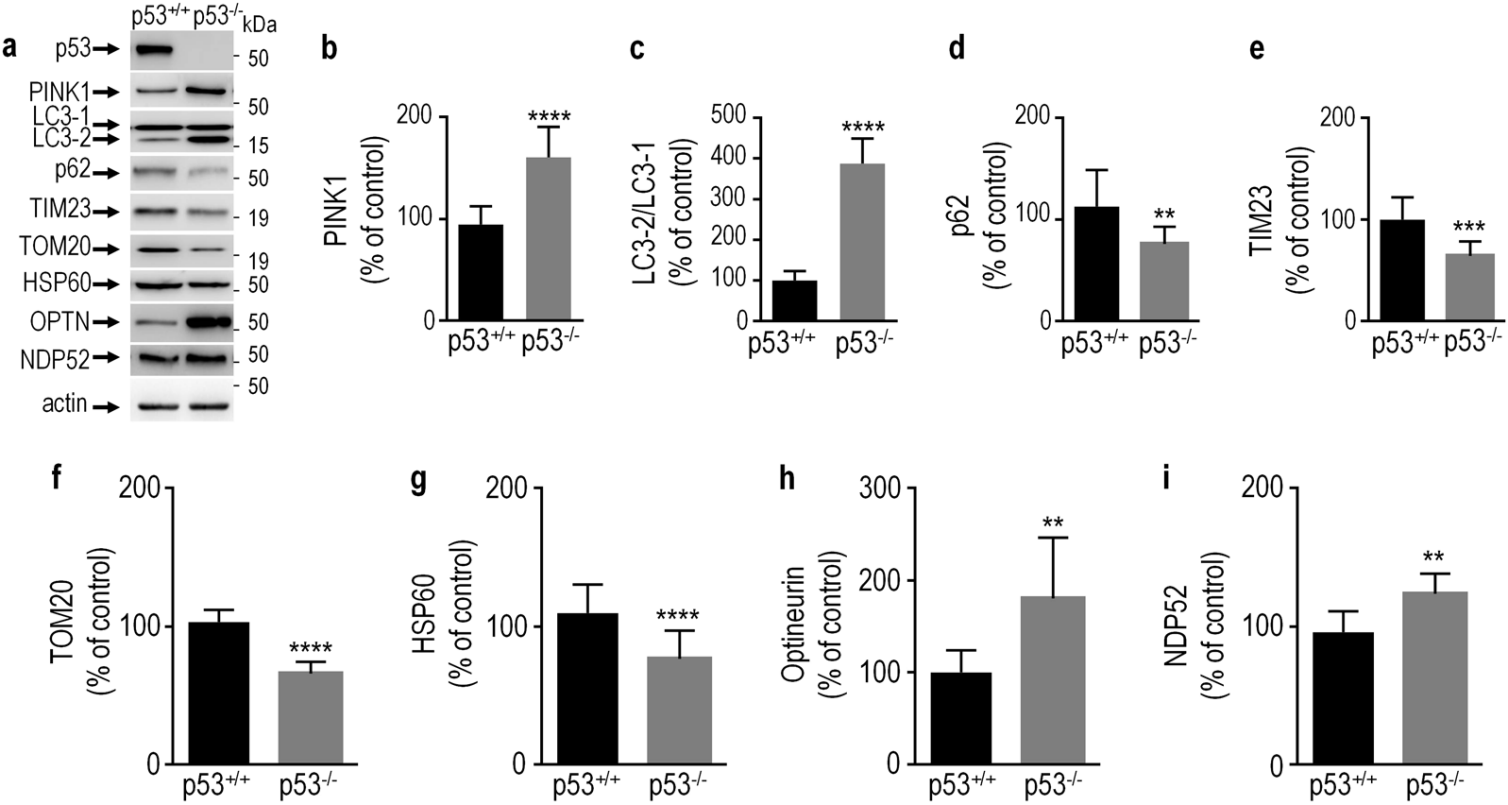
*TP53* knockout triggers a pro-mitophagic response. PINK1 (**a**,**b**, *N* = 8), LC3-2/LC3-1 (**a**,**c**, *N* = 8), p62 (**a**,**d**, *N* = 12), TIM23 (**a**,**e**, *N* = 10), TOM20 (**a**,**f**, *N* = 9), HSP60 (**a**,**g**, *N* = 16), optineurin (**a**,**h**, *N* = 9) and NDP52 (**a**,**i**, *N* = 8) protein levels were analyzed in control (p53^+/+^) or *TP53*-deficient (p53^−/−^) HAP1 cells as described in the Methods. Bars represent the means ± SEM of 3-4 independent experiments performed in triplicate and are expressed as percent of control (p53^+/+^) cells. Actin expression is provided as a representative gel loading control in **a**. Statistical analyses were performed with GraphPad Prism software by using unpaired Student’s *t*-test. Significant differences are: ** *p* < 0.01, ***p < 0.001 and ****p < 0.0001.

**Fig. 7:**
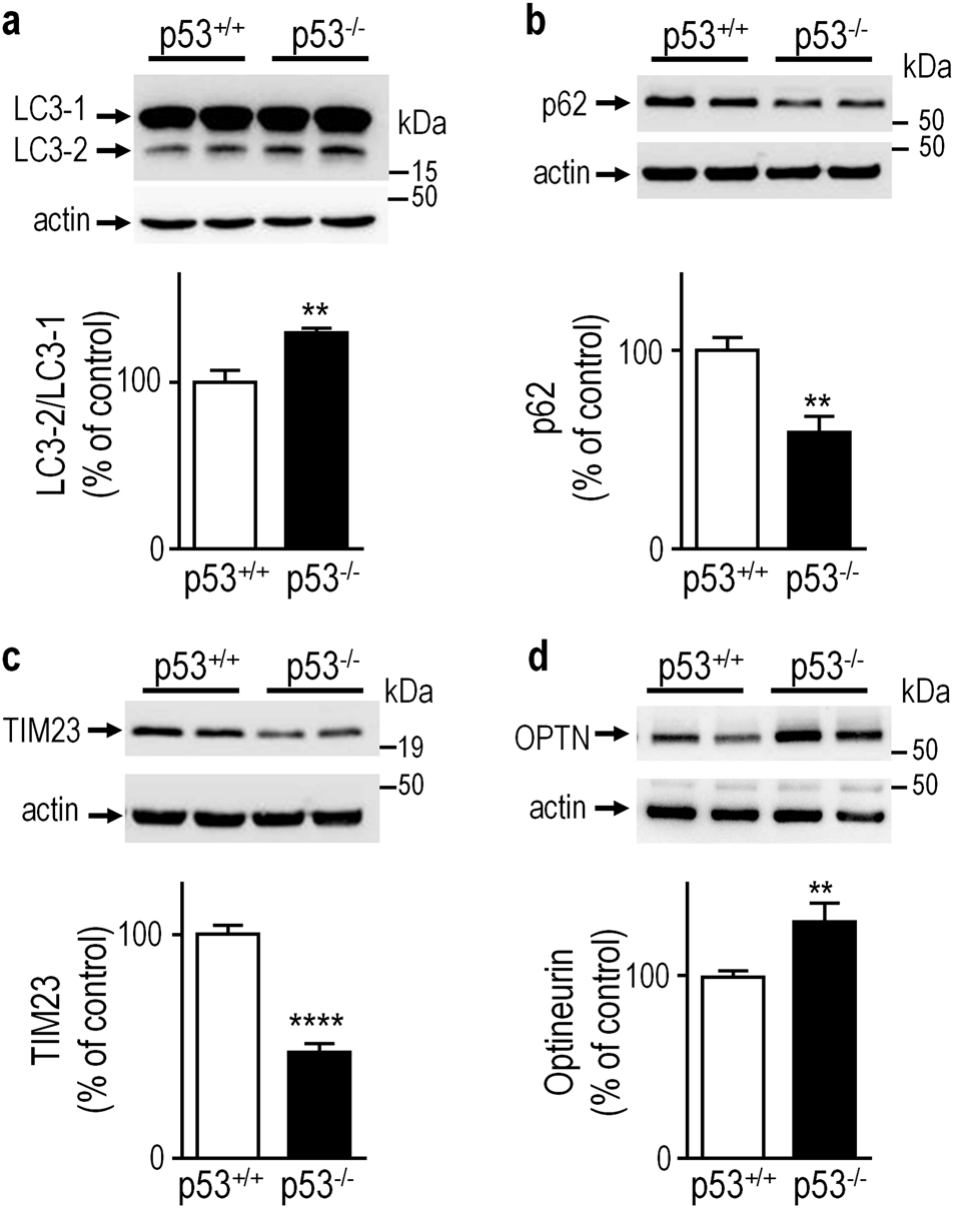
Evaluation of mitophagy control by p53 in mice brain. LC3-2/LC3-1 (**a**, *N* = 9), p62 (**b** *N* = 9), TIM23 (**c**, *N* = 9) and optineurin (OPTN, **d**, *N* = 9) protein levels were analyzed in control (p53^+/+^) or *TP53*-deficient (p53^−/−^) knockout mice brains as described in the Methods. Bars represent the means ± SEM of 3 independent experiments performed in triplicate and are expressed as percent of control (p53^+/+^) cells. Actin expression is provided as a representative gel loading control in **a**. Statistical analyses were performed with GraphPad Prism software by using unpaired Student’s *t*-test. Significant differences are: ** *p* < 0.01, ***p < 0.001.

The pro-mitophagy response of PINK1 is associated to increased LC3 maturation, decreased expression of mitophagy markers like TIM23, TOM20 and HSP60 and modulation of p62, optineurin and NDP52 autophagy receptors [25–27]. From our data indicating a p53-mediated repression of *PINK1*, one should expect that *TP53* invalidation mimics PINK1-related pro-autophagy response. As shown in Fig. 6, full *TP53* invalidation (see abolishment of p53 expression in 6**a**) increases PINK1 expression levels (Fig. 6a, b), LC3 maturation (Fig. 6a, c) optineurin (Fig. 6a, h) and NDP52 expressions (Fig. 6a, i) while it decreases the levels of p62 (Fig. 6a, d) and the mitophagy markers TIM23 (Fig. 6a, e), TOM20 (Fig. 6a, f) and HSP60 (Fig. 6a, g). These observations fully mimic the PINK1-associated pro-mitophagy phenotype previously described. Importantly, *TP53* knockout mice brain analysis corroborates this conclusion. Thus, Fig. 7 shows that *TP53* invalidation increased levels of LC3-2 (Fig. 7a) and optineurin (Fig. 7d) and reduced p62 (Fig. 7b) and TIM23 (Fig. 7c) expressions. In addition, we have also carried out ShRNA inactivation of TP53 by transient transfection in SH-SY5Y cells. Our data indicate that in the neuronal dopaminergic cell system, again p53 modulates p62, HSP60; TIM23 and TOM20 similarly to HAP cells and mice brain (data not shown).

The control of mitophagy is tightly linked to PINK1 and parkin interplay. Thus, parkin itself represses *TP53* [9], indirectly increases *PINK1* transactivation [25] and is controlled by p53 [10, 11]. This intricate functional network led us to question the putative implication of parkin in the regulation of PINK1 by p53. We have treated control and parkin knockout cells with PFT and evaluated the influence of this p53 transcriptional inhibitor on *PINK1* mRNA levels. In agreement with our previous studies [9, 25], we confirmed that parkin deficiency lowers *PINK1* mRNA levels (compare black bars in Supplementary Fig. S2a) and increases p53 mRNA levels (compare black bars in Supplementary Fig. S2b). However, PFT did not prevent parkin-mediated *PINK1* regulation (compare grey bars in Supplementary Fig. S2a) thus indicating that *PINK1* regulation by p53 is parkin independent.

### Negative control of mitophagy by p53 is dependent of PINK1

**Fig. 8:**
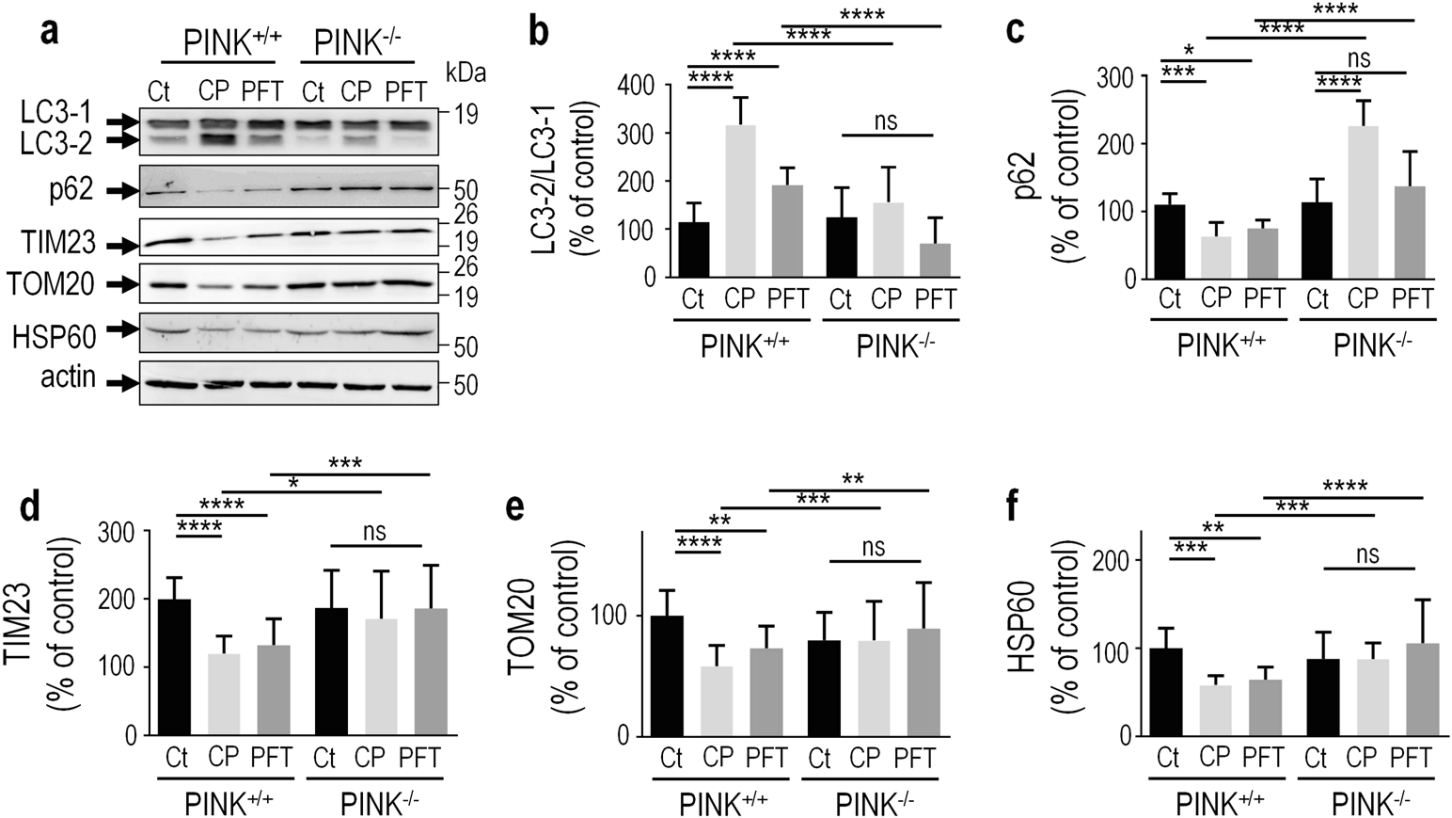
p53 repression of autophagy is PINK1-dependent. LC3-2/LC3-1 (**a**,**b**, *N* = 12), p62 (**a**,**c**, *N* = 12), TIM23 (**a**,**d**, *N* = 12), TOM20 (**a**,**e**, *N* = 12) and HSP60 (**a**,**f**, *N* = 12) protein levels were analyzed in control (PINK^+/+^) or *Pink1*-deficient (PINK^−/−^) HAP cells treated with vehicle (Ct), pifithrin-α (PFT, 30 µM, 4 h) or CCCP (CP, 10 µM, 6 h) as described in the Methods. Bars represent the means ± SEM of 4 independent experiments performed in triplicates and are expressed as percent of control HAP1 (HAP^+/+^, Ct) cells. Actin expression is provided as a representative gel loading control in **a**. Statistical analyses were performed with GraphPad Prism software by using unpaired Student’s *t*-test. Significant differences are: *ns* not significant, *p < 0.05, ** *p* < 0.01 and ***p < 0.001.

The similar phenotypes triggered by TP53 invalidation (Figs. 6 and 7) and PINK1 expression [25–27] strongly support our view of a p53-dependent control of mitophagy via *PINK1* repression. In order to definitely confirm this hypothesis, we have assessed the contribution of PINK1 to p53-linked mitophagic phenotype. Thus, we treated human haploid (HAP) cells harboring (PINK^+/+^) or lacking (PINK^−/−^) endogenous PINK1 with PFT. First, in agreement with previous data [7, 8] the blockade of endogenous p53 by PFT increases autophagic markers similarly to CCCP (CP), the mitochondrial uncoupler usually used to unravel PINK1 mitophagy response. Thus, PFT and CP-treated PINK1^+/+^ cells display increased expressions of LC3-2 (Figs. 8a, b) and decreased levels of p62 cargo protein (Figs. 8a, c) and mitochondrial markers TIM23 (Figs.8a, d), TOM20 (Figs. 8a, e) and HSP60 (Figs. 8a, f) confirming that pharmacological impairment of p53 activity leads to a pro-mitophagy phenotype. As expected PINK1^−/−^ cells show a decrease of LC3 maturation (Figs. 8a, b), concomitant to an increase of p62 (Figs. 8a, c), TIM23 (Figs. 8a, d), TOM20 (Figs. 8a, e) and HSP60 (Figs. 8a, f) protein levels when compared to PINK1^+/+^ cells in stress (CP and PFT) conditions. Of most interest, PFT-mediated effect on these autophagic/mitophagic markers was fully abolished by *PINK1* depletion (Fig. 8a-f). This data firmly comfort our hypothesis of PINK1-dependent p53-associated repression of mitophagy.

## Discussion

Several works underlined a duality in the ability of p53 to control autophagic response [7, 8, 28]. Thus, both p53 activation and depletion trigger increased autophagy. It is consensually admitted that genotoxic stress or oncogenic activation triggers a p53-dependent pro-autophagic phenotype that is mostly due to nuclear p53 transcriptional activity [3, 4]. Conversely, p53-linked anti-autophagic response was reported to be independent of its ability to regulate gene transcription but rather linked to cytoplasmic p53. Thus, it was shown that several autophagic inducers enhanced the proteasomal degradation of p53 and thereby, down-regulate the p53-mediated anti-autophagic phenotype [7]. Furthermore, the work of Tasdemir et al. has demonstrated that endogenous p53 could repress autophagy via the inhibition of AMPK and activation of mTOR [7]. This conclusion was supported by the observation that p53, in which nuclear export domain was mutated, exclusively resides in the nucleus and was unable to repress autophagy. Overall, this work proposed that the p53 anti-autophagic effect is exclusively linked to its cytoplasmic localization [7]. Our work however indicates that the situation is likely not so simplistic. Thus, we clearly demonstrated by combined and complementary approaches that p53 can also repress autophagy, *ex-vivo* and *in vivo*, via the transcriptional repression of *PINK1*, a well characterized pro-autophagic effector [29]. Thus, nuclear p53 can also trigger an anti-autophagic phenotype. The discrepancy between our work and that of Tasdemir’s may be linked to the fact that these authors investigated the impact of nuclear p53 on “non-selective” macro-autophagy and several players of the AMPK/mTOR axis. Our data show that nuclear p53 may thus have an additional role on PINK1-dependent control of “selective” mitochondrial autophagy. Furthermore, since transcriptional regulation may be cell type specific, one cannot exclude the possibility that in Tasdemir et al., the use of p53-deleted human colon adenocarcinoma cells could have precluded the delineation of additional p53-dependent transcriptional pathways repressing autophagy.

Several lines of evidence indicate that mitophagy could well be controlled by an intricate functional interplay linking parkin, p53 and PINK1. First, there exists a feed-back loop by which, parkin transrepresses p53 [9], which conversely, up-regulates parkin transcription [11]. This interplay has functional consequences where PINK1 plays a key role. Thus, cytoplasmic p53 affects parkin translocation to the mitochondria, enabling its interplay with PINK1 and thereby, elimination of malfunctioning mitochondria by mitophagy [30]. Numerous studies have documented the functional relationship between parkin and PINK1. This led to the consensus that parkin acts as a privileged downstream effector of a cascade by which PINK1 controls mitochondrial homeostasis and elimination of flawed mitochondria by selective autophagy [31]. Breaking this dogma, we have recently shown that parkin could also act upstream to PINK1 to regulate its transcription and pro-mitophagic function [25]. Thus, one could envision a cascade in which p53 repression by parkin would ultimately lead to PINK1 up-regulation. However, we demonstrate here that PINK1 regulation by p53 is independent of parkin, thus suggesting the existence of an alternative interplay between these proteins controlling mitophagy. This scenario where PINK1 could modulate mitophagy independently of parkin has been demonstrated in previous works (for review see 27). Overall, this data highlight multiple pathways by which p53 could control mitophagy, including yet unraveled additional function of nuclear p53 as an autophagic repressor via transcriptional repression of *PINK1*.

Since p53 expression and function are altered in both neurodegenerative diseases and cancer and given the fact that both pathologies are characterized by drastic alterations of autophagic processes, one can envision that either gain (neurodegeneration) or loss (tumorigenicity) of function of p53 could directly influence PINK1 function and thereby, could contribute to the anatomical stigmata and clinical pictures observed in these pathologies.

## Materials and methods

### Cellular and animal models

Mouse embryonic fibroblasts (MEF), Human Colorectal adenocarcinoma (HCT116), SH-SY5Y human neuroblastoma and human embryonic kidney (HEK293) cell lines were cultured in Dulbecco’s modified Eagle’s medium supplemented with 10% fetal calf serum, penicillin (100 U/ml) and streptomycin (50 µg/ml) purchased from Life Technologies (CA, USA) and incubated at 37°C in a 5% CO_2_ atmosphere. Immortalized mouse embryonic fibroblasts invalidated for *TP53* or for *TP53* and *p19*^*arf*^ genes were kindly provided by Dr. M. Roussel (St. Jude Children’s Research Hospital Memphis, TN, USA) whereas the human colorectal carcinoma cell lines HCT116 invalidated or not for *TP53* were provided by Dr. JC Bourdon (University of Dundee, Dundee, UK). MEF cells invalidated for the *PARK2* (parkin) gene were kindly provided by Dr. T. Dawson (Johns Hopkins University School of Medicine, Baltimore, MD, USA.). HAP1 cell line, purchased from Horizon Genomics (UK), is a haploid human cell line that was derived from KBM-7 cells [32]. HAP1 clones HZGHC000798c008 (HAP PINK1^−/−^) and HZGHC001068c001 (HAP p53^−/−^) were engineered using CRISPR/cas9 approach. HAP1 cells were cultured in Iscove’s Modified Dulbecco’s Medium (IMDM) with 10% fetal calf serum, penicillin (100 U/ml) and streptomycin (50 µg/ml) purchased from Life Technologies (CA, USA). *TP53* knockout mice have been provided by Dr. M. Serrano (Spanish National Cancer Research Center, Madrid, Spain). Human cell lines from ATTC were validated by STR profiling method according to manufacturer’s instruction (geneprint® 10 system, Promega, France) and routinely tested for mycoplasma contamination.

### Pharmacological modulation of p53

The pharmacological modulation of p53 was obtained after incubations with etoposide (16 h, 150 µM), pifithrin-α (4–16 h, 10–30 µM) leptomycin (16 h, 10 nM) purchased from Sigma (MO, USA). The pharmacological modulation of PINK1 was obtained after 6 h incubations with CCCP (10 µM) purchased from Sigma. After treatments, cells were recovered and protein and RNA analyses were monitored as described below. For the analysis of the impact of these drugs to *PINK1* promoter activity, cells were transiently transfected with the promoter of PINK1 driving the luciferase reporter gene expression (see Luciferase-based reporter assays section) and 24 h post-transfection submitted to the above described treatment.

#### Immunohistochemical analysis

SH-SY5Y cells were cultured on glass coverslips in 35 mm dishes, transiently transfected with empty vector, wild type or mutated p53 cDNA by means of lipofectamine and then fixed with 1.5% *p*-formaldehyde for 20 min, washed three times in PBS and incubated with anti-p53 CM1 antibodies for two hours. After three additional washes with PBS, cells were incubated with goat anti-rabbit secondary antibody conjugated to Alexa Fluor-488 (Molecular Probes, MA, USA) for 1 h. 300 nM DAPI (Roche Diagnostics S.A.S, France) was added (with PBS in order to stain the nuclei. Coverslips were then mounted in Vectashield mounting medium (Vector Laboratories LTD, UK) and staining was visualized with a confocal microscope. The same protocol was used when cells were cultured on glass coverslips in 35 mm dishes and treated with vehicle (DMSO) or leptomycin (10 nM, 16 h).

### Western-blot protein analysis

Cells and mouse brains were lysed in Prusiner’s buffer (Tris-HCl 1M; pH 7.5 containing NaCl (150 mM), EDTA (5 mM), Triton X100 (0.5%), deoxycholate and protease inhibitor cocktail) and the homogenates obtained briefly sonicated. Aliquots of 50 µg of total protein were loaded to (8–16%) SDS-PAGE gels. After migration, proteins were wet-transferred to Hybond C membranes (GE Healthcare Europe GmbH, France) and immunoblotted using the following antibodies: anti-PINK1 C-terminal (BC100-494, Novus Biologicals, France) and (AC-R3173-2, Abiocode, CA,USA); anti-p53 (CM1, kindly provided by J.C. Bourdon); anti-LC3 (NB100-2220, Novus Biologicals); anti-p62/SQSTM1 (NBP1-49956, Novus Biologicals); anti-TIM23 (611222, BD Biosciences, USA); anti-TOM20 (612278, BD Biosciences, USA), anti-HSP60 (sc-59567, Santa Cruz Biotechnology Inc., Germany), anti-optineurin (sc-166576, Santa Cruz Biotechnology Inc., Germany), anti-NDP52 (sc-376540, Santa Cruz Biotechnology Inc., Germany) and anti-actin (clone AC-74, A5316, Sigma) antibodies. Immunological complexes were revealed with either anti-rabbit or anti-mouse IgG-coupled peroxidase antibodies (Jackson ImmunoResearch, UK) by the electrochemiluminescence detection method (Roche Diagnostics S.A.S, France). Chemiluminescence was recorded using a luminescence image analyzer LAS-4000 (Raytest, Fuji, France) and quantifications of non-saturated images were performed with the FUJI Film Multi Gauge image analyzer software.

### Plasmid constructs and ex-vivo transfection

Human and mouse full length and mouse 5′end-truncated PINK1 promoter-luciferase constructs have been previously described [33]. The pGL3 vector containing the mouse *PINK1* promoter served as a template to generate the promoter deleted of the 5′-CCAG-3′ nucleotides. This deleted motif constitutes part of the 5′end half-site of p53 putative binding site, 5′-***CCAG***ctgcac***CAAG***-3′, located from nucleotides -167 to -154 upstream of mPINK1 ATG start codon. The primers used were 5′- GGT TCA AAG TGC AAA CTG CAC CAA GGG ATG -3′ (forward primer) and 5′- CAT CCC TTG GTG CAG TTT GCA CTT TGA ACC -3′ (reverse primer). The plasmids GFP-p53 and GFP-p53 NES were a gift from Tyler Jacks (Addgene plasmids # 12091 and 12092). We used the GFP-p53 vector to generate with the mutagenesis kit QuickChange II (Stratagene, CA, USA) the GFP-p53NLS mutant (mutation of the 280-282 KKK sequence of p53 nuclear localization signal (NLS) in AAA). The following forward and reverse mutagenesis primers: 5′-TCC TCT CCC CAG CCA GCG GCG GCA CCA CTG GAT GGA GAA TAT-3′ and 5′-ATA TTC TCC ATC CAG TGG TGC CGC CGC TGG CTG GGG AGA GGA-3′ purchased from Eurogentec (France) were used. All the constructs were verified by full sequencing. Transient transfections of the various cells systems cells were carried out by means of lipofectamine 2000 (Life Technologies) according to the manufacturer’s instructions.

### Luciferase-based reporter assays

The transactivation of the wild-type (human and mouse) and mutated *PINK-1* mouse promoter described in the plasmid constructs section was followed by recording the luciferase reporter gene activity 24 h after co-transfection of 1 μg of the above cDNAs and 0,5 µg of β-galactosidase cDNA (in order to normalize for transfection efficiencies) by means of Luciferase and β-galactosidase enzyme assays systems according to the manufacturer’s (Promega) instructions. When necessary, in a subset of experiments, 0,5–1 µg of empty pcDNA3.1, wild type or mutated p53 were co-transfected.

### RNA extraction, reverse transcription and real-time PCR analysis

RNA from cells and RNA later (Qiagen, Germany) stabilized mouse brains were extracted and treated with DNAse using RNeasy or RNeasy Plus Universal Mini kits respectively following manufacturer’s instructions (Qiagen). Two µg of total RNA were reverse transcribed (GoScript Reverse Transcriptase, Promega) using Oligo-dT priming. Then, samples were subjected to real-time PCR by means of a Rotor-Gene 6000 apparatus (Qiagen), using the SYBR Green detection protocol. Gene-specific primers were designed with the Universal Probe Library Assay Design Center software (Roche Applied Science, France). Relative expression levels of human *PINK-1* (forward 5′-CGA GGA ACT CGT TTG AAG GG-3′; reverse 5′-CCA GGT GGC AAA TCA GAC ATG-3′), mouse *PINK-1* (forward 5′-CGC CTA TGA AAT CTT TGG GC-3′; reverse 5′-GCA CTG CCT TGG CCA TAG AA-3′), amplification products were normalized for RNA concentrations with human *Topoisomerase 1* (forward 5′-CCC TGT ACT TCA TCG ACA AGG-3′; reverse 5′-CCA CAG TGT CCG CTG TTT C-3′), mouse *Topoisomerase 1* (forward 5′-TGC CTC CAT CAC ACT ACA GC-3′; reverse 5′-CGC TGG TAC ATT CTC ATC AGG-3′) and human *GAPDH* (forward 5′-AGC CAC ATC GCT CAG ACA C-3′; reverse 5′-GCC CAA TAC GAC CAA ATC C-3′) and mouse *GAPDH* (forward 5′-TGT CCG TCG TGG ATC TGA C-3′; reverse 5′-CGT GCT TCA CCA CCT TCT TG-3′) housekeeping genes.

### Chromatin immunoprecipitation (ChIP) assay

We performed ChIP according to EZ- ChIP kit instructions (Millipore). Briefly, 10^7^ cells were fixed with formaldehyde (1% final concentration), treated with glycine to quench unreacted formaldehyde and recovered in cold phosphate buffered saline (PBS) containing the protease inhibitor cocktail II. Pelleted cells were resuspended in the SDS lysing buffer and sonicated on ice in order to yield chromatin fragments of about 200–500 bp in size. After a pre-clearing step using protein G Agarose, immunoprecipitation was performed with either anti-p53 primary antibody (CM1, JC Bourdon, University of Dundee, Dundee, UK) or normal mouse IgG as a negative control. Immuno-complexes were then incubated with a solution of protein G-agarose. After elution of the immuno-complexes from beads and crosslinks reversal RNAse and proteinase K treatments aimed at eliminating remaining RNAs and proteins were performed. DNA was purified and subjected to a real-time PCR using primers (forward: 5′-GTT-GTT-CAC-AAC-CCC-TCG-ACC-TGG-G-3′; reverse: 5′-GAC-AAC-AAC-AAA-CTT-CGG-GGG-CGG-C-3′) specific for the -205/-47 DNA region (159 bases-long amplicon) upstream the start codon of mouse *PINK1* gene. We calculated the fold enrichment (FE) as the ratio of the amplification efficiency of the ChIP sample over that of the IgG (FE = % ChIP/% IgG). Briefly, to do so we first produced a standard curve by performing qPCR with *PINK1* primers on known (10-fold serial dilutions) DNA quantities of input DNA. We then did qPCR runs of ChIP and IgG samples along with the dilution series of the input DNA standards. We created a linear regression plot with the Ct (Threshold Cycle) obtained and plot Ct versus DNA quantity (log scale) of the dilutions to generate the slope of the standard curve. We calculated the efficiency of the primers [formula: E (%efficiency) = 10(−1/slope)-1] and the amplification efficiency (AE) [formula: AE = 10(−1/slope)]. We calculated the % ChIP [formula: % ChIP = AE (Input Ct-ChIP Ct)*(dilution factor) (100)] and the % IgG [formula: % ChIP = AE(Input Ct-IgG Ct)*(dilution factor) (100)]. This classical procedure allows calculating fold enrichment (FE) [formula: FE = % ChIP/% IgG].

### Adenovirus-mediated p53 overexpression in mouse brain

C57BL6 mice were purchased from Charles River Laboratories (France) and maintained at 21°C on a 12 h light and 12 h dark cycle. Animal care procedures were in accordance with the guidelines established by the European Community Council directives (86/609/EEC). C57BL6 adult males were deeply anesthetized with ketamine (100 mg/kg of body weight; Ketamine 1000; Ceva) and xylazine (10 mg/kg of body weight; Rompun 2%; Centravet) dissolved in 0.9% sterile saline, and positioned in a stereotaxic frame. Eight animals (male, 3 months-aged mice) per group were injected into the striatum, using appropriate coordinates (1 mm anterior to the bregma, 2 mm lateral, 3 mm deep from the skull surface) with either eGFP adenovirus or adenovirus expressing human p53 and GFP (10^7^ PFU in each hemisphere; Vector Biolabs, Philadelphia, PA, USA). Animals were sacrificed 10 days after injection. Proteins and mRNA were extracted and analyzed as described above. We did Western-blot analysis with the p53 CM1 antibody to show the expression of the injected p53 and GFP.

### Statistical analysis

Statistical analyses were performed with GraphPad Prism software (www.graphpad.com for Windows, CA, USA). No statistical test was used to determine sample size in ex vivo and in vivo studies. No randomization and blindness were used in animal studies. Analysis of two groups of variables was used for samples analyzed by unpaired Student’s t-test after having passed a normality test (D’Agostino-Pearse omnibus Normality test) to assure Gaussian distribution of values. All tests are two-sided; the mean was defined as the center value and errors bars correspond to SEM. Analysis of more than two groups of variables (normality test passed) was performed by One-way ANOVA with Newman-Keuls’s post-hoc test without any adjustment. The number of samples, replication of experiments and the P value are provided in figures legends.

## Electronic supplementary material

Suppl. Figs and legends

expanded blots
