# Supplementary material for: Nuclear p53-mediated repression of autophagy involves PINK1 transcriptional down-regulation
Source: Cell Death Differ. 2018 Jan 19;25(5):873–84. doi: 10.1038/s41418-017-0016-0 (PMC5943347; doi:10.1038/s41418-017-0016-0)
Supplement: Supplementary file 1 — Suppl. Figs and legends [file 41418_2017_16_MOESM1_ESM.doc]

**p53-mediated repression of autophagy involves PINK1 transcriptional regulation.**

**Goiran et al.**

**Figure S1. Impact of p53 knockdown to *PINK1* transcription.**

Control mouse fibroblasts (MEF, p19^arf-/-^, A-, white bars) or MEF lacking *TP53* (p19^arf-/-p53-/-^, AP-, black bars) were assessed for *PINK1* promoter transactivation (**a, N=9**) and mRNA levels (**b, N=9**) in basal conditions as described in the Methods section. Bars represent the means ± SEM of 3 independent experiments performed in triplicates and are expressed as percentage of control cells taken as 100 (A-, white bars). Statistical analyses were performed with GraphPadPrism software by using unpaired Student’s t-test. Significant differences are: **p< 0.01.

**Figure S2. Impact of Parkin knockdown to *PINK1* transcription regulation by p53.**

Mouse fibroblasts harboring (PK^+/+^) or lacking endogenous parkin (PK^-/-^) were assessed for *PINK1* (**a, N=9**) and p53 mRNA levels (**b, N=9**) in basal (DMSO, black bars) or pifithrin-α-treated (PFT, gray bars) conditions as described in the Methods. Bars represent the means ± SEM of 3 independent experiments performed in triplicates and are expressed as percentage of control cells (PK^+/+^) taken as 100. Statistical analyses were performed with GraphPadPrism software by One-way ANOVA with Newman-Keuls’s post-hoc test without any adjustment. Significant differences are: *p<0.05, **p< 0.01, ***p<0.001 and ****p<0.0001.
