# Supplementary material for: Nuclear p53-mediated repression of autophagy involves PINK1 transcriptional down-regulation
Source: Cell Death Differ. 2018 Jan 19;25(5):873–84. doi: 10.1038/s41418-017-0016-0 (PMC5943347; doi:10.1038/s41418-017-0016-0)
Supplement: Supplementary file 2 — expanded blots [file 41418_2017_16_MOESM2_ESM.pdf]

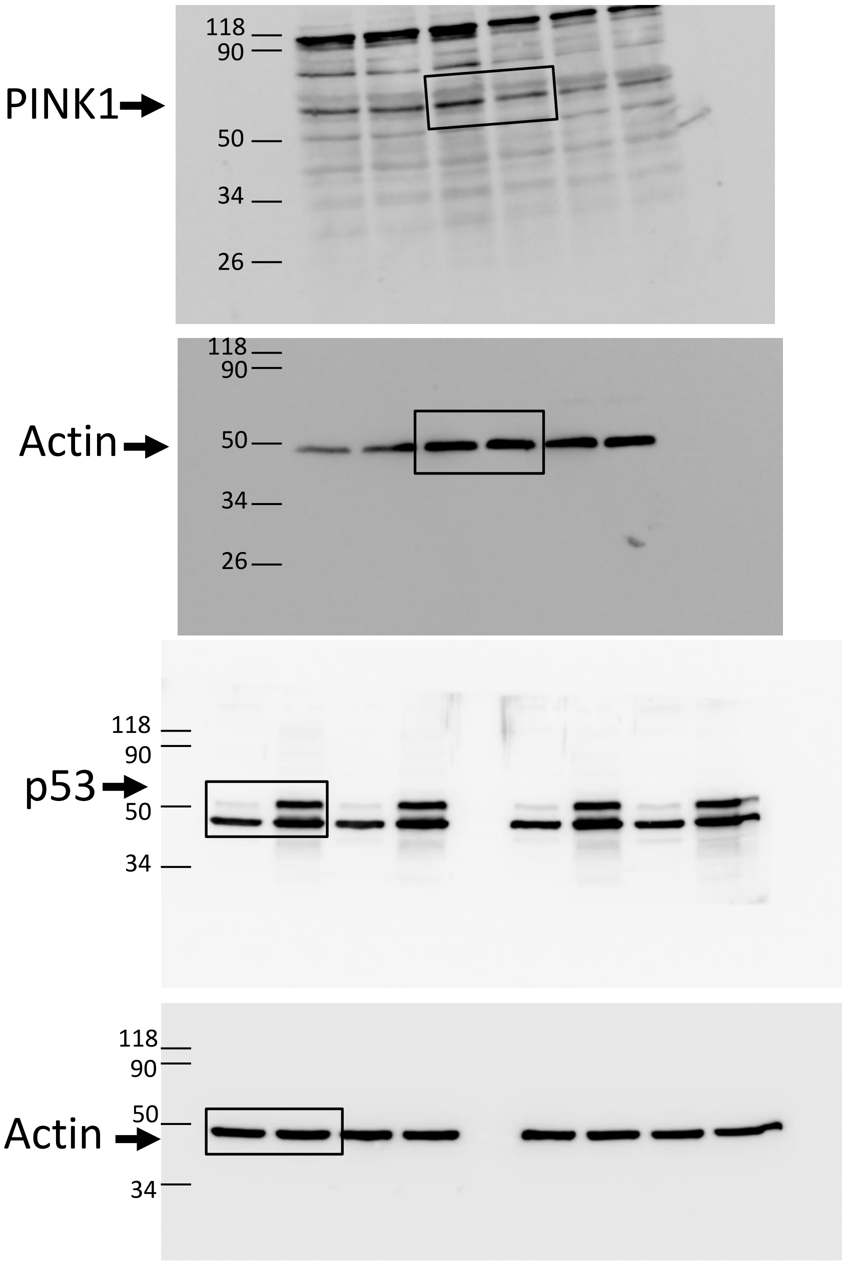

Expanded blots 1a

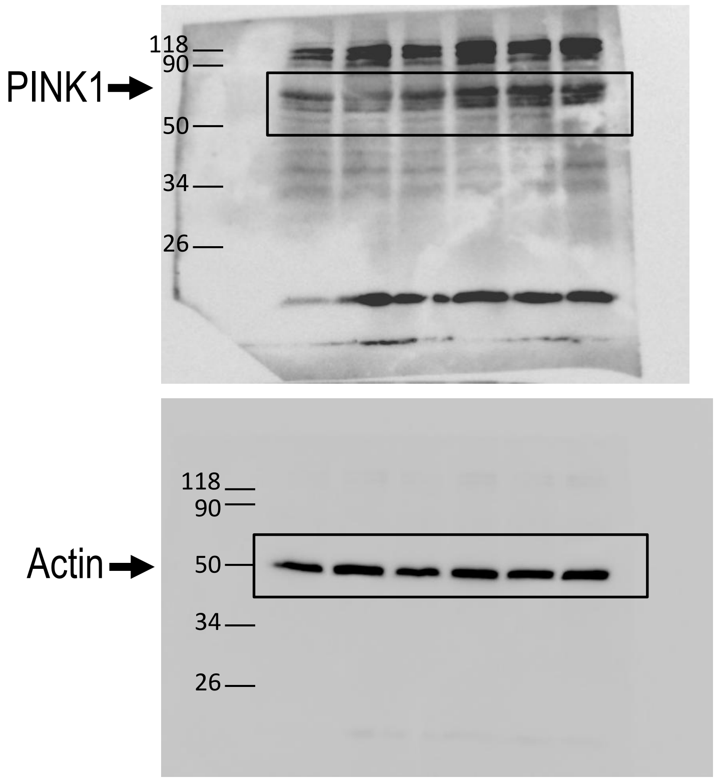

Expanded blots 1d

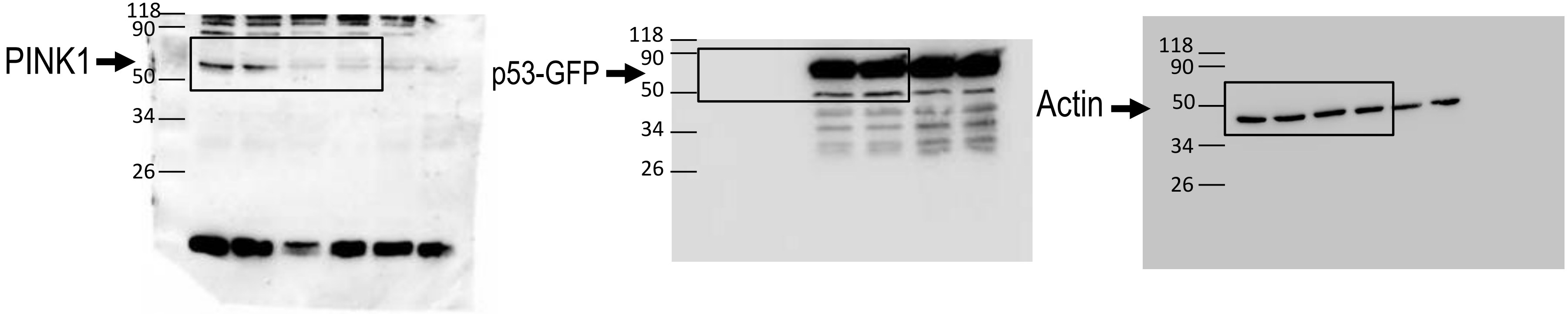

Expanded blots 2a

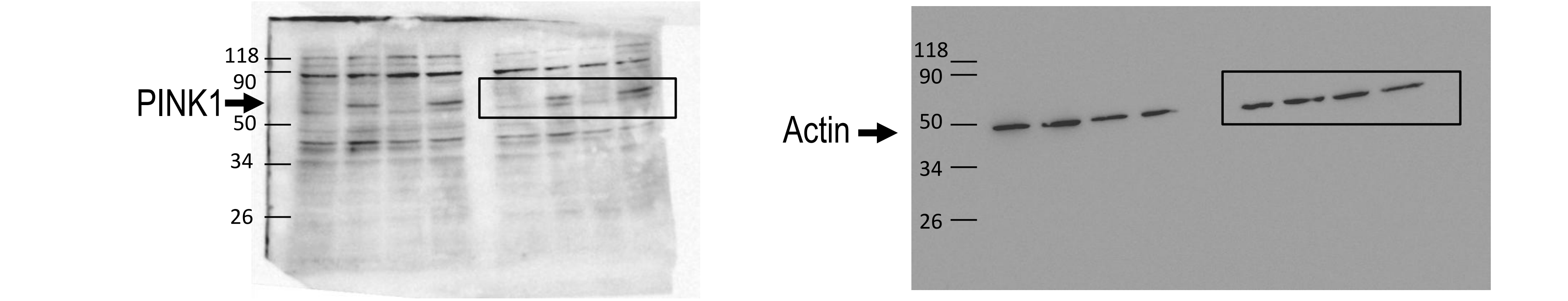

Expanded blots 2d

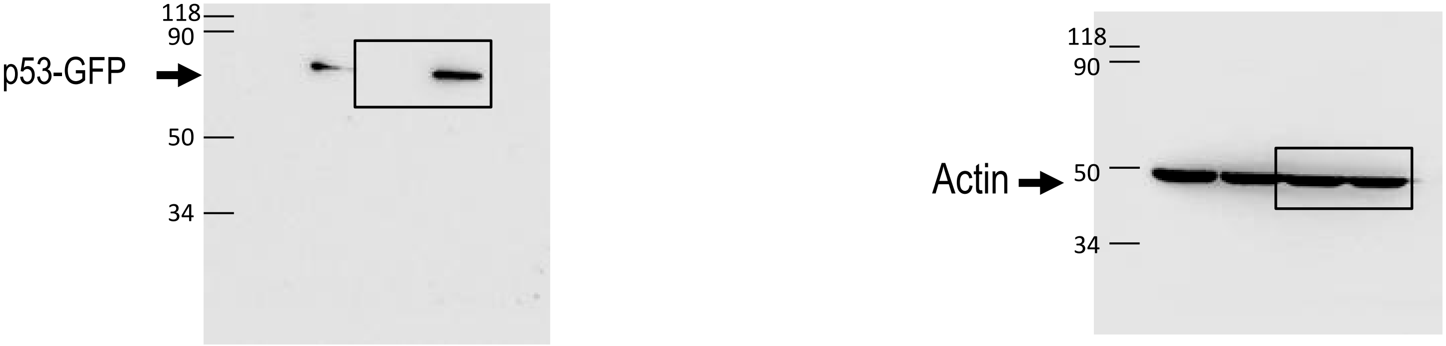

Expanded blots 2g

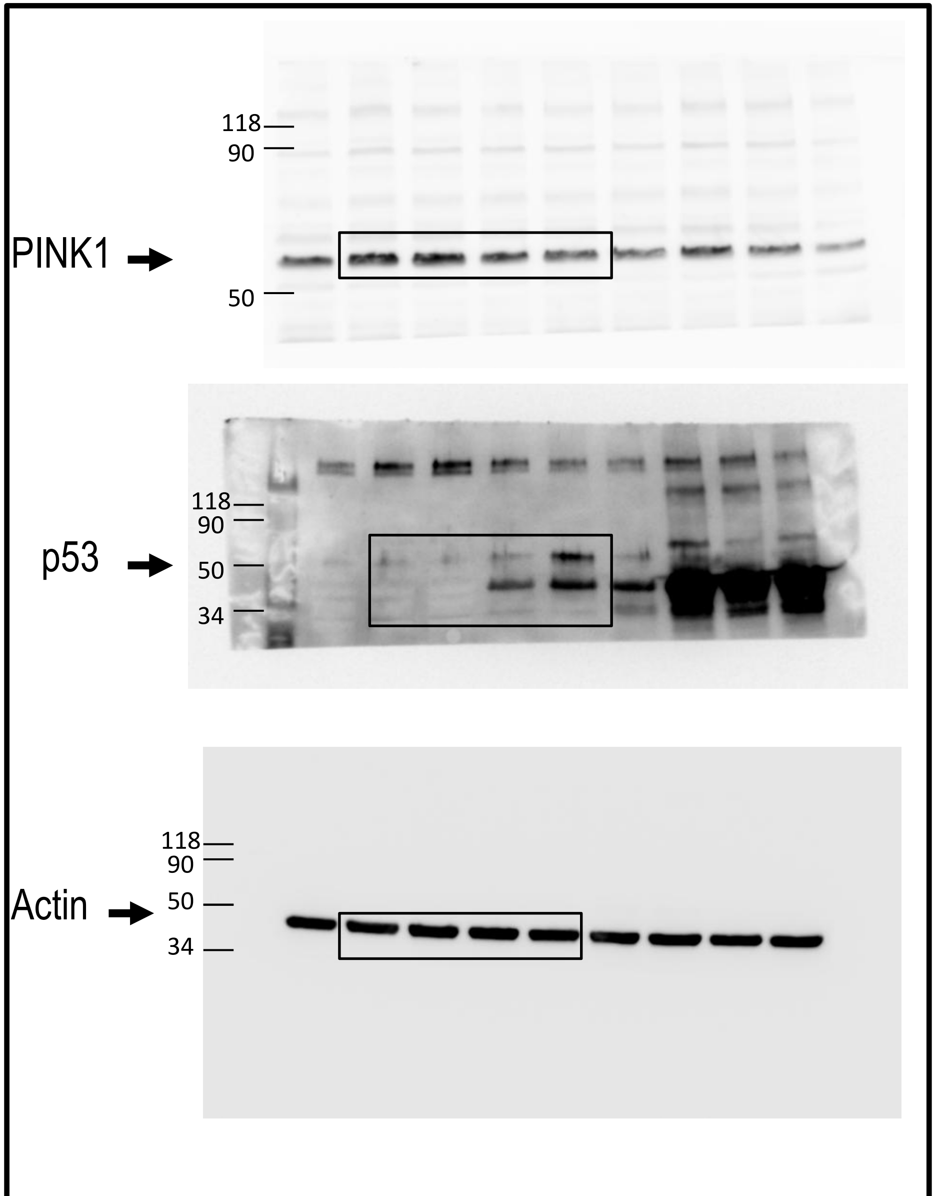

Expanded blots 3a

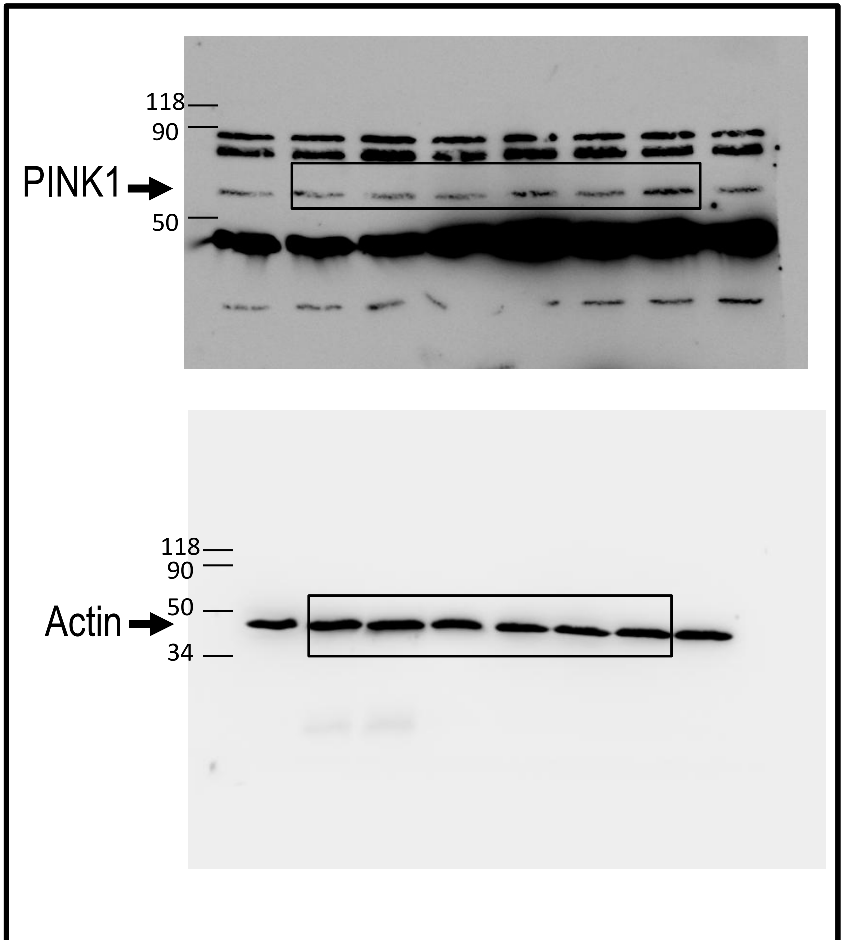

Expanded blots 3c

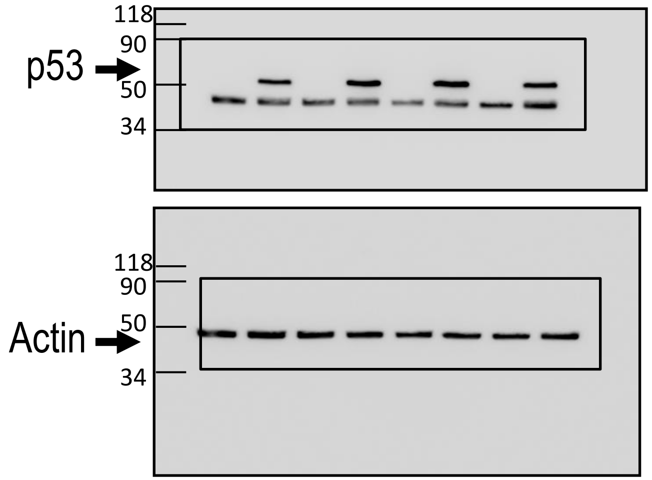

Expanded blots 4a

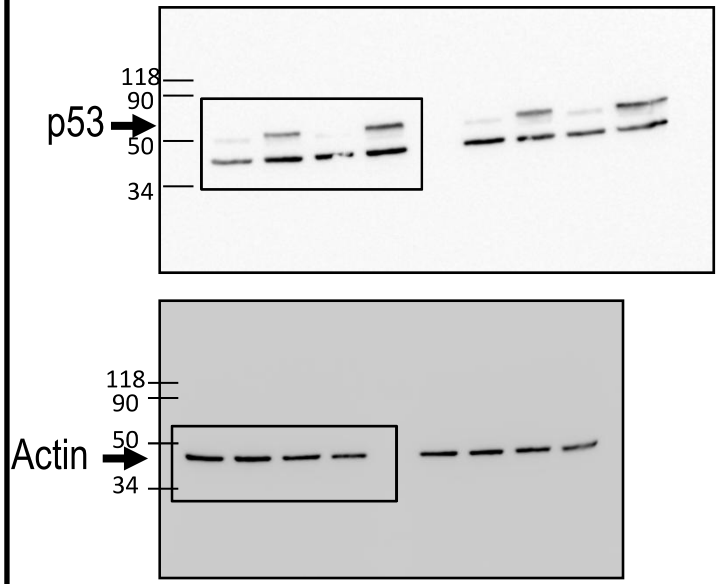

Expanded blots 4b

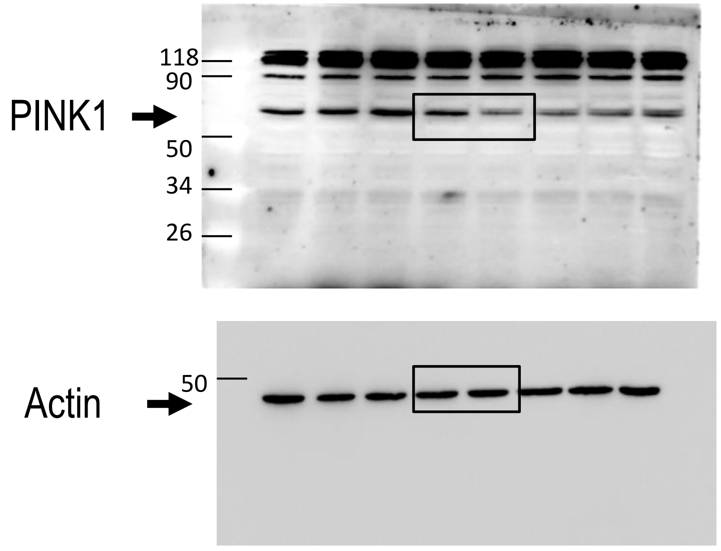

Expanded blots 5b

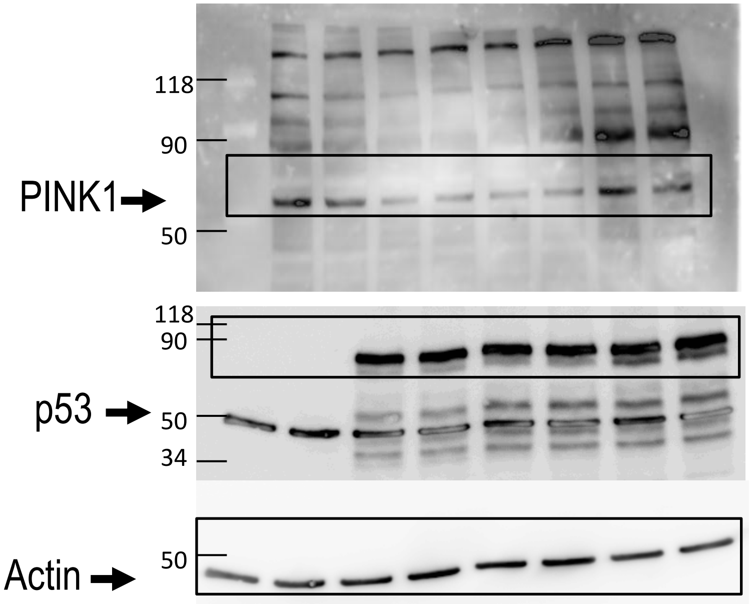

Expanded blots 5f

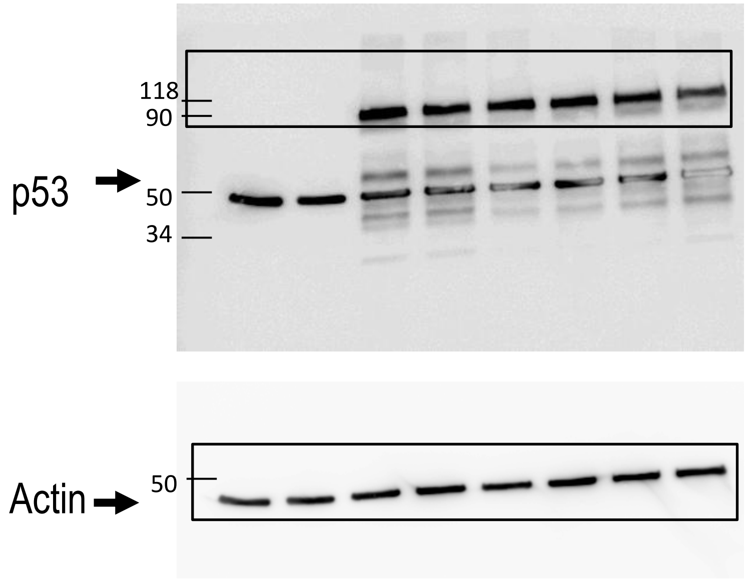

Expanded blots 5g

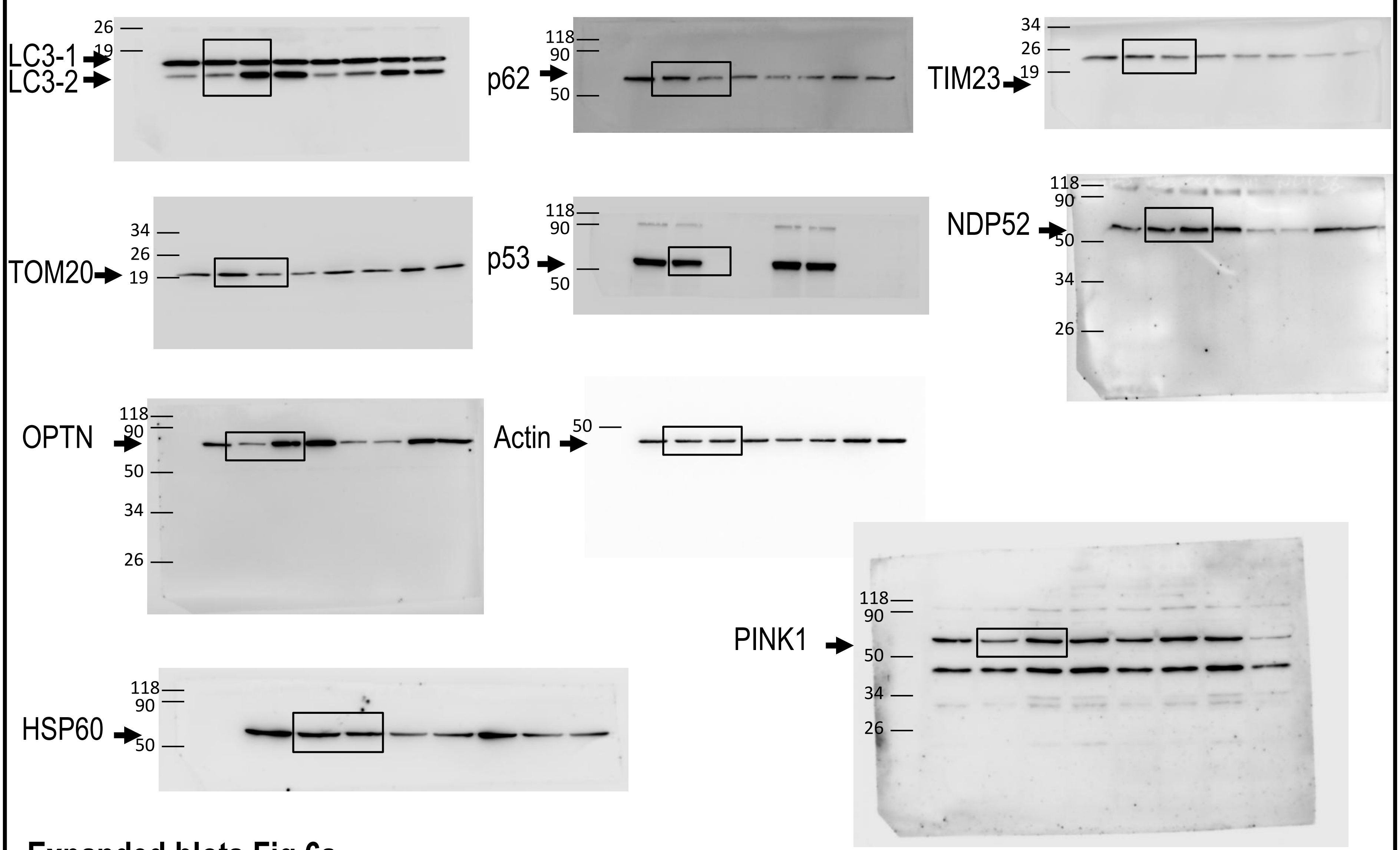

Expanded blots Fig.6a

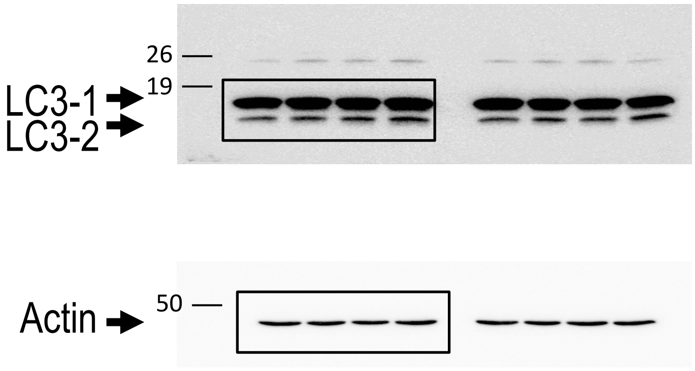

Expanded blots Fig.7a

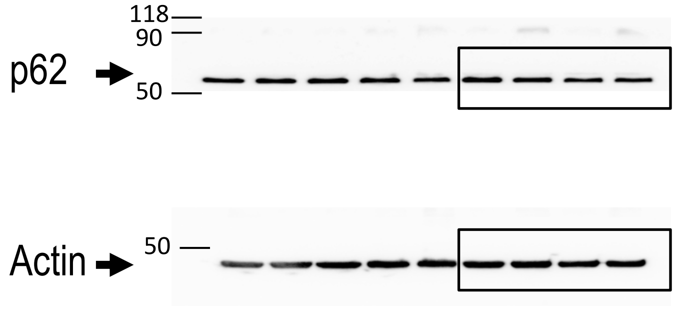

Expanded blots Fig.7b

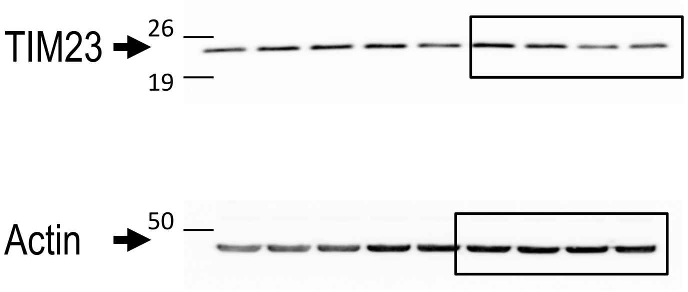

Expanded blots Fig.7c

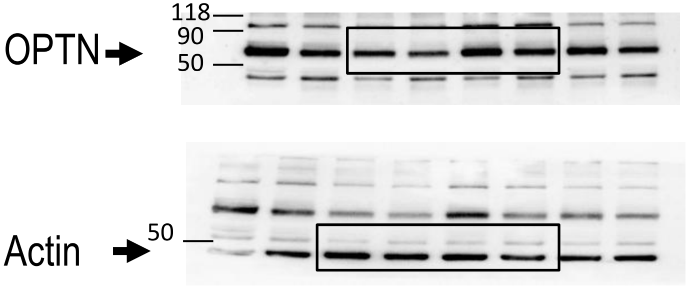

Expanded blots Fig.7d

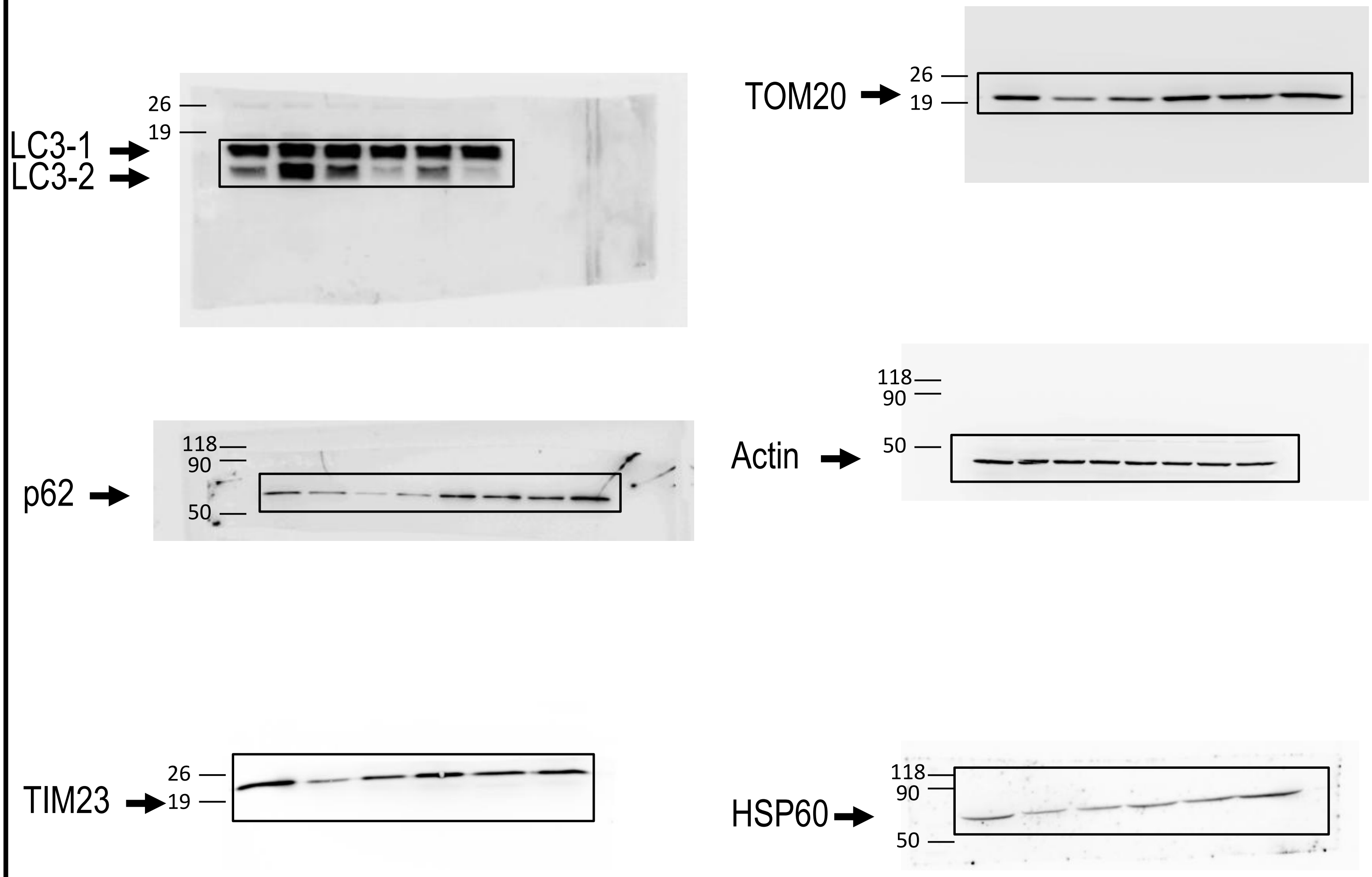

Expanded blots Fig.8a
